# Measurement and Reporting of Oxidative Stress and Antioxidant Biomarkers in Exercise Intervention Studies in Adults with Cancer: A Scoping Review

**DOI:** 10.3390/antiox15101280

**Published:** 2026-10-03

**Authors:** Hae Sung Lee, Jong-Shik Kim

**Affiliations:** 1 Department of Physical Education, College of Education, Wonkwang University, 460, Iksan-daero, Iksan 54538, Jeonbuk, Republic of Korea; haesung2@wku.ac.kr; 2 Department of Sports Science, College of Life Care, Wonkwang University, 460, Iksan-daero, Iksan 54538, Jeonbuk, Republic of Korea

**Keywords:** oxidative stress, exercise oncology, physical activity, cancer survivors, redox biomarkers, methodological heterogeneity, scoping review

## Abstract

Oxidative stress has been implicated in both cancer pathogenesis and treatment-related toxicity, and structured exercise has been proposed as a non-pharmacological strategy that may help modulate this redox imbalance in patients and survivors with cancer, although the consistency of this effect across the existing literature remains unclear. This scoping review, conducted in accordance with PRISMA-ScR guidance, searched four databases for exercise intervention studies reporting at least one oxidative stress or antioxidant biomarker in adult cancer patients or survivors, published between January 2000 and July 2026. Data were charted narratively rather than pooled, with attention to biomarker category, biological specimen, analytical method, sampling timing, and the completeness of exercise-prescription and treatment-context reporting. Twelve reports representing ten independent cohorts met the eligibility criteria. The evidence base consisted of randomized, non-randomized, and single-arm studies and was dominated by studies involving women with breast cancer. Because nine of the 12 reports enrolled women with breast cancer, the findings primarily characterize this population and may not be directly generalizable to other cancer types, male patients, or patients receiving different treatment regimens. Across the included reports, reductions in lipid peroxidation products and markers of protein or DNA oxidative damage were reported in some studies, whereas findings for enzymatic antioxidants and composite redox indices were directionally inconsistent. However, these descriptive patterns should be interpreted cautiously because the included studies varied substantially in design, specimen type, analytical platform, sampling timing relative to exercise and treatment, and reporting completeness. Overall, the heterogeneity in study design, treatment context, biomarker measurement, and sampling procedures precludes conclusions regarding the magnitude or direction of exercise-related redox effects in this population.

## 1. Introduction

Oxidative stress, defined as an imbalance between the production of reactive oxygen species (ROS) and the capacity of enzymatic and non-enzymatic antioxidant systems to neutralize them, is recognized as a contributing mechanism in tumor initiation and progression through DNA damage, lipid peroxidation, and dysregulated redox-sensitive signaling [1]. This biochemical disturbance is not confined to carcinogenesis itself; several classes of anticancer treatment, including anthracyclines, platinum compounds, and ionizing radiation, have been shown to further elevate ROS production and markers of oxidative DNA damage such as 8-hydroxy-2′-deoxyguanosine (8-OHdG), an effect that appears to persist beyond the immediate treatment period in a subset of survivors [2,3]. Because malignancy and its treatment converge on overlapping redox pathways, oxidative stress and antioxidant status have increasingly been proposed as candidate biomarkers for monitoring treatment tolerability (including cardiotoxicity) and long-term survivorship outcomes in oncology populations [4]. However, the clinical utility of any single marker for this purpose remains to be firmly established [5].

Structured exercise has been investigated as a non-pharmacological intervention that may influence this redox balance in patients with cancer, but its relationship with oxidative stress is not unidirectional. A single bout of exercise can transiently increase ROS generation in contracting skeletal muscle, and this acute pro-oxidant stimulus has been proposed as one potential trigger for subsequent upregulation of endogenous antioxidant enzymes such as superoxide dismutase and glutathione peroxidase within a hormetic framework [6]. However, it remains unclear whether this adaptive response is preserved in patients undergoing active cancer treatment, whose baseline redox and metabolic status may already be altered, and the magnitude of the hormetic response appears to vary considerably between individuals depending on training status, treatment phase, and possibly genetic factors [7]. This interindividual variability, together with differences in specimen type, assay method, and the timing of sample collection relative to both the exercise bout and the treatment cycle, may partly explain why redox outcomes reported in exercise oncology studies are not always consistent in direction or magnitude [6,8].

The biological context of active cancer treatment may further modify the capacity to mount or detect an exercise-related redox adaptation. Cytotoxic chemotherapy and radiotherapy can increase reactive oxygen species generation as part of their therapeutic action, while tumor burden, systemic inflammation, reduced nutritional intake, and treatment-related toxicities may also contribute to mitochondrial dysfunction and oxidative stress in skeletal muscle and other tissues [9,10]. These treatment-related influences may alter baseline redox status, exercise tolerance, and the temporal pattern of biomarker responses. In addition, the interaction between exercise and anticancer treatment is unlikely to be uniform across tumor types, treatment modalities, or treatment phases [11]. Thus, an observed change in a circulating redox biomarker during active treatment cannot be assumed to reflect exercise-related adaptation alone and should be interpreted in relation to the concurrent treatment regimen, timing within the treatment cycle, recent exercise exposure, and the biological compartment represented by the biomarker.

Two prior systematic reviews have synthesized evidence on exercise and oxidative stress biomarkers in adults with cancer, generally reporting that structured exercise is associated with improvements in total antioxidant capacity and reductions in malondialdehyde or 8-OHdG concentrations, although the certainty of these findings was limited by small sample sizes and considerable heterogeneity across the included trials [12,13]. Both reviews were primarily oriented toward summarizing the direction of intervention effects, and neither was designed to systematically characterize how these biomarkers were operationalized across studies, that is, which biological specimens were sampled, which analytical platforms were used, at what time points relative to exercise and treatment samples were collected, and how completely these methodological details were reported. Because differences in biomarker measurement approaches may complicate meta-analytic pooling and cross-study comparison, this methodological dimension of the literature has, to our knowledge, not yet been mapped independently of an effectiveness-oriented synthesis.

The present study therefore adopts a scoping review design rather than a systematic review or a meta-analysis one, given its aim to characterize the breadth and pattern of measurement and reporting practices rather than to estimate pooled effect sizes or appraise intervention efficacy. Specifically, this review addresses the following questions: (1) which categories of oxidative stress and antioxidant biomarkers have been assessed in exercise intervention studies among adult patients and survivors with cancer; (2) through which biological specimens and analytical methods have these biomarkers been measured, and at which time points relative to exercise sessions and cancer treatment; and (3) how completely have exercise-prescription variables and treatment-related contextual factors been reported alongside these biomarker outcomes. Therefore, by mapping these characteristics rather than pooling their effects, this review aims to provide a methodological foundation that may inform the design and reporting of future exercise oncology trials incorporating redox biomarkers. Given the anticipated predominance of breast cancer studies and the limited number of reports available for other tumor types and treatment settings, this review was not designed to estimate cancer-specific or treatment-specific effects of exercise on redox biomarkers.

## 2. Materials and Methods

### 2.1. Protocol and Registration

This scoping review was conducted in accordance with the methodological framework originally proposed by Arksey and O’Malley [14] and subsequently refined by Levac et al. [15] and is reported following the Preferred Reporting Items for Systematic Reviews and Meta-Analyses extension for Scoping Reviews (PRISMA-ScR) checklist [16]. Given that the current review does not aim to appraise or pool intervention effects but rather to map the measurement and reporting characteristics of a specific biomarker domain, a scoping review design was considered more appropriate than a conventional systematic review, consistent with published guidance on selecting between the two approaches [17]. An a priori protocol specifying the research questions, eligibility criteria, search strategy, and data charting form was developed before the literature search and was registered on the Open Science Framework (OSF) on 21 August 2026 (https://osf.io/9hd2q/).

### 2.2. Eligibility Criteria

Eligibility criteria were defined using the Population–Concept–Context (PCC) framework recommended for scoping reviews [18], as summarized in Table 1.

### 2.3. Information Sources and Search Strategy

A systematic search was conducted across four electronic databases: PubMed/MEDLINE, Embase, Scopus, and the Web of Science Core Collection. These databases were selected to ensure broad coverage of the biomedical, oncology, and exercise science literature, and the search covered the period from 1 January 2000 to 1 July 2026. The search strategy combined controlled vocabulary (MeSH terms in PubMed, Entree terms in Embase) with free-text keywords across three concept blocks, cancer, exercise, and oxidative stress/antioxidant biomarkers, connected using Boolean operators. The full database-specific search strategies, including controlled vocabulary and free-text terms used at the time of the search, are presented in Table 2.

### 2.4. Study Selection Process

**Figure 1:**
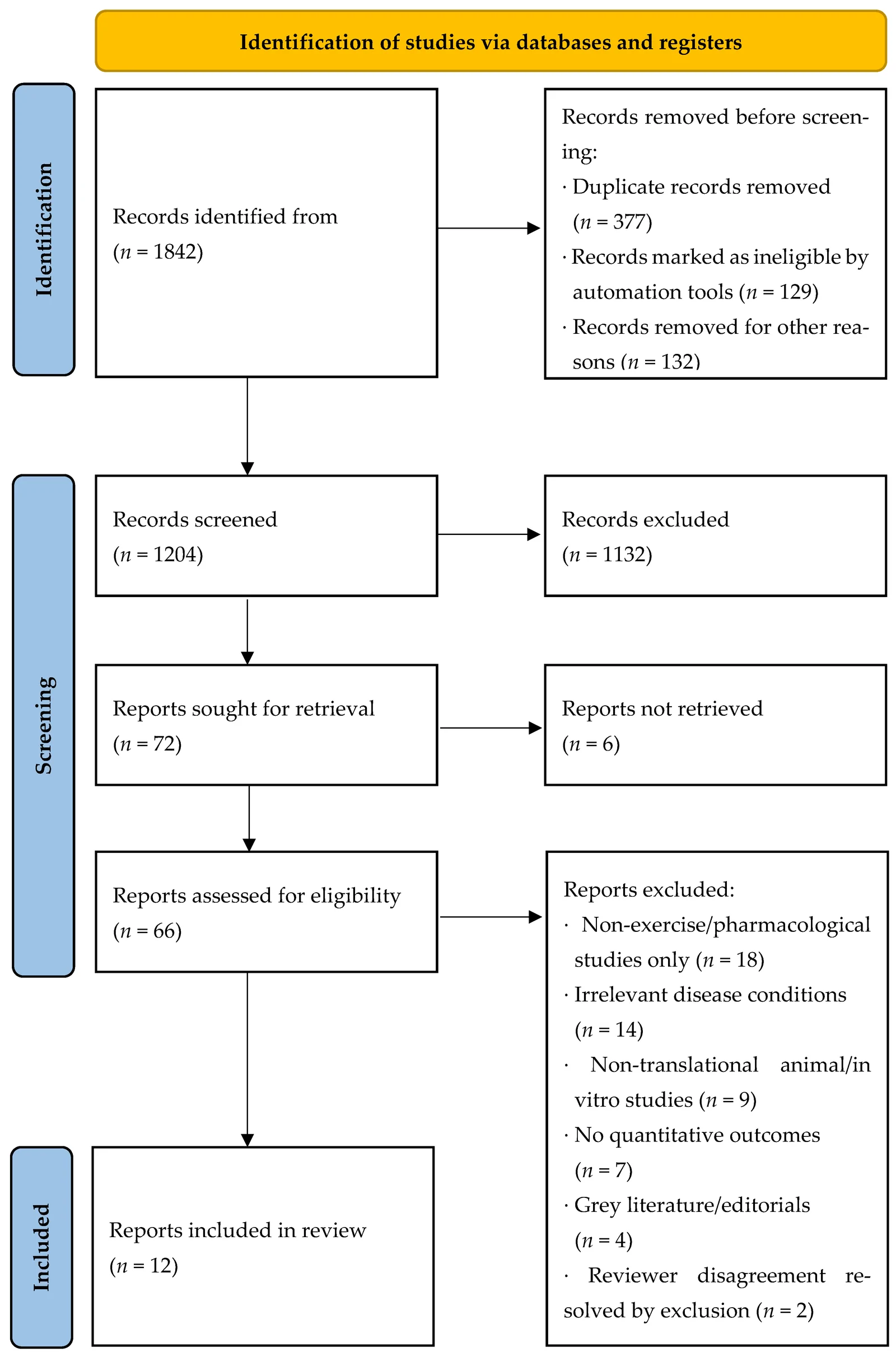
Study selection and screening protocol following PRISMA-ScR guidelines.

All records retrieved from the four databases were imported into a reference-management platform, and duplicate records were identified and removed before screening. Title and abstract screening, followed by full-text assessment of potentially eligible articles, was performed independently by two reviewers. Disagreements at either the title/abstract or full-text stage were resolved through discussion between the two reviewers. Before formal screening, the reviewers pilot-tested the eligibility criteria on a sample of records and refined the operational definitions of structured exercise interventions and eligible redox biomarkers. The final eligibility criteria were applied consistently during title/abstract screening and full-text assessment. The study selection process is presented in a PRISMA-ScR flow diagram (Figure 1), including records identified from each database, records identified through citation searching where applicable, records removed before screening, records screened, reports sought for retrieval, reports assessed for eligibility, and reports excluded after full-text assessment with prespecified reasons.

### 2.5. Data Charting Process and Data Items

The following data items were extracted from each included report: (1) author, year, and country; (2) cancer type, disease stage, and sex distribution; (3) treatment phase at the time of the exercise intervention and the reported treatment regimen; (4) sample size, study design, and cohort identity where multiple reports arose from the same trial; (5) exercise prescription characteristics, including modality, frequency, intensity, session duration, program length, progression, supervision, and adherence where reported; (6) the prespecified redox biomarker category, classified as lipid peroxidation, oxidative DNA damage, protein oxidation, enzymatic antioxidant defense, non-enzymatic antioxidant defense, total oxidant/antioxidant capacity, or nitrosative stress; (7) the biological specimen and fraction used for biomarker assessment; (8) the analytical method and normalization approach where reported; (9) the timing of sample collection relative to the most recent exercise session and, where applicable, the cancer treatment cycle; and (10) the direction of the reported statistical comparison or within-group change, as presented by the original authors, recorded descriptively without calculation or pooling of effect estimates. Data were charted independently by two reviewers using a piloted data charting form. Discrepancies in extracted information were resolved through discussion, with reference to the full text of the original report. When multiple reports arose from the same cohort, they were retained as separate reports only when they contributed distinct redox outcomes or measurement information; cohort identity was recorded to avoid treating such reports as independent studies in the narrative synthesis.

### 2.6. Critical Appraisal

Consistent with current methodological guidance for scoping reviews, a formal critical appraisal of methodological quality or risk of bias was not undertaken, as the objective of this review is to characterize the extent and pattern of the available evidence rather than to evaluate the strength or certainty of intervention effects. The distribution of study designs across the included literature is instead reported descriptively in the Results section, providing an indirect indication of the overall composition of the evidence base without constituting a formal quality appraisal.

### 2.7. Synthesis of Results

No meta-analysis was undertaken because the purpose of this scoping review was to map measurement and reporting practices rather than to estimate intervention efficacy. Extracted data were synthesized narratively and organized according to biomarker category, biological specimen, analytical method, sampling timing, exercise prescription, and treatment context. Counts of reports were used only to describe the distribution of methodological characteristics across the evidence base. The direction of biomarker findings was summarized as reported by the original studies but was not treated as a pooled estimate, comparative effect, or indication of the superiority of any exercise modality, biomarker, or treatment setting.

## 3. Results

The database search, conducted from 1 January 2000 to 1 July 2026, identified 1842 records across PubMed/MEDLINE, Embase, Scopus, and the Web of Science Core Collection. Before screening, 638 records were removed, including 377 duplicate records, 129 records marked as ineligible by automation tools, and 132 records removed for other reasons. The remaining 1204 records were screened by title and abstract according to the PCC-based eligibility criteria. Of these, 1132 records were excluded because they did not involve adult patients or survivors with cancer, did not evaluate a structured exercise intervention, or did not report an oxidative stress or antioxidant biomarker outcome. Seventy-two reports were sought for retrieval, of which six could not be retrieved. The remaining 66 full-text reports were assessed for eligibility. Fifty-four reports were excluded after full-text assessment because they evaluated non-exercise or pharmacological interventions only (*n* = 18), involved populations or disease conditions outside the eligibility criteria (*n* = 14), reported non-human or in vitro research (*n* = 9), did not report eligible quantitative biomarker outcomes (*n* = 7), were gray literature or editorials (*n* = 4), or did not meet other prespecified eligibility criteria (*n* = 2). Twelve reports met the eligibility criteria and were included in the data charting process; these reports represented ten independent cohorts. The included reports comprised randomized controlled trials, a non-randomized multi-group comparison, a quasi-experimental study with sequential group allocation, and single-arm pre–post intervention studies conducted among adults with breast cancer, head and neck cancer, or colorectal cancer.

### 3.1. General Characteristics of Included Studies

Of the 12 included reports, nine enrolled women with breast cancer [19,20,21,22,23,24,25], whereas the remaining reports addressed mixed solid-tumor survivors [26], head and neck cancer during chemotherapy [27], and colorectal carcinoma following primary treatment [28]. Sample sizes ranged from 12 to 130 participants [21,25], with most studies enrolling fewer than 50 and at least two explicitly framed as pilot or feasibility work [19,25]. Study designs varied across reports and included randomized controlled trials [23,24], non-randomized comparative studies [27], quasi-experimental designs [20], and single-arm pre--post studies [20,25,29,30]. Five reports, representing four cohorts, were conducted during active systemic therapy or radiotherapy [19,22,23,24,27]. The remaining reports were conducted from the immediate post-treatment period to longer-term survivorship; however, the treatment phase was not reported consistently across all studies. The timing of exercise interventions ranged from concurrent chemotherapy, radiotherapy, endocrine therapy, or trastuzumab treatment to the post-treatment survivorship period; however, treatment status and treatment cycle information were not reported consistently across studies. Two pairs of publications derived from the same underlying trials—Delrieu et al. [29,30] (ABLE trial), and Jain et al. [23,24]—and were charted separately owing to distinct outcome sets, accounting for the difference between 12 reports and 10 cohorts. Because several reports were derived from the same underlying cohorts, study design and cohort identity were considered separately during data charting (Table 3).

### 3.2. Exercise Intervention Characteristics

Program duration, modality, frequency, and session structure varied considerably across the 12 studies, ranging from a 2-week inpatient comparison to a 48-week yoga-based intervention [23,24,28], with most remaining programs falling within a 6- to 16-week window [20]; full study-level parameters are presented in Table 3. Exercise intensity was described using markedly heterogeneous and largely non-interchangeable metrics, which limits direct comparison of the physiological stimulus applied across trials [19,20,21,28]. Several studies prescribed intensity as a percentage of age-predicted maximal heart rate or heart rate reserve [19,20], one used a percentage of one-repetition maximum following a within-week undulating scheme [20], and others relied on target ratings of perceived exertion or an individualized daily step count target without a heart rate- or load-based equivalent [21,30]. Neither the moderate-versus-high-intensity comparison in colorectal cancer nor the pilot endurance-training study specified intensity using a metric directly comparable to the heart rate- or load-based approaches used elsewhere [25,28].

With respect to supervision, most interventions were delivered under direct or remote real-time supervision by a qualified exercise professional, whereas two self-administered or step count-based programs relied primarily on periodic feedback [19,21]. Adherence was quantified in a subset of studies, with completion rates generally exceeding 75–85% among retained participants [19,20,23]; comparable figures were not consistently reported elsewhere, restricting broader inference about program feasibility across this literature.

### 3.3. Categories of Oxidative Stress and Antioxidant Biomarkers Assessed

The 12 included studies collectively assessed biomarkers spanning eight broad categories of redox and related inflammatory function, although no single study addressed all categories simultaneously, and the reported direction of change was not uniform even within a given category.

Lipid peroxidation products, most commonly malondialdehyde (MDA) [20,24,29] or thiobarbituric acid-reactive substances (TBARS) [19,25], were assessed in seven studies [19,20,21,22]. Reductions in MDA or TBARS were reported in some studies [19,20,21,24], whereas other reports described no statistically significant change [22,25,27,29,30]; in one of the latter studies [29], an association between elevated MDA and sarcopenic status was observed at the six-month follow-up; however, the intervention itself was not associated with a statistically significant change in MDA concentration. Oxidative DNA damage was assessed using plasma 8-OHdG or urinary 8-oxo-dG in several reports [26,27,28,30]. A significant reduction in urinary 8-oxo-dG was reported following moderate-intensity exercise in the colorectal cancer study [28], and a reduction in plasma 8-OHdG was reported after supervised exercise training in patients with head and neck cancer [27]. By contrast, in the single-arm ABLE trial of women with metastatic breast cancer, plasma 8-OHdG did not change significantly from baseline to the six-month follow-up [30]. Because this study used a single-arm design, the reported within-group change does not provide a controlled estimate of the effect of the walking intervention on oxidative DNA damage.

Enzymatic antioxidants, principally superoxide dismutase (SOD) [19,20,22,24,30], catalase (CAT) [19,20,22,24,30], and glutathione peroxidase (GPx) [20,21,30], showed heterogeneous responses across the included reports [19,20,21,22,24,30]. In the ABLE single-arm trial, plasma GPx activity increased significantly by 17% after the six-month individualized walking intervention, whereas plasma SOD and CAT activities did not change significantly [30]. In this single-arm study, changes in plasma GPx activity were positively correlated with changes in physical activity level and daily step count, whereas changes in leucocyte NOX activity were inversely correlated with changes in physical activity and fitness [30]. A comparative study further reported that SOD and CAT activities were significantly higher in the supervised-exercise arm than in either the non-exercising breast cancer group or the healthy reference group [22], while two remaining studies found no significant between-group differences in these enzymes [20,24]. Non-enzymatic antioxidant capacity, including total or reduced glutathione [19,22], and total free thiols [19], was assessed in four studies [19,20,22,30].

Composite indices of total oxidant or antioxidant status—Trolox-equivalent antioxidant capacity (TAC) [19,26,27,29], ferric-reducing antioxidant power (FRAP) [25], total oxidant status (TOS) [22], and the oxidative stress index (OSI; the TOS-to-TAC ratio) [22]—were reported in six studies [19,22,25,26,27,29]. A significant post-intervention increase in antioxidant capacity was observed in two of these [19,27], while no significant change was found in three others [25,26,29]. In the remaining study [22], TAC, TOS, and OSI were significantly higher in the supervised-exercise group than in both comparison groups. Protein oxidative damage markers, including protein carbonyls [22,26,27,29] and advanced oxidation protein products (AOPP) [22,30], were assessed in five studies [22,25,26,27,29], with a significant post-training reduction reported in two [26,27] and no significant net change reported in a third [29]; in the remaining two studies, carbonyl or AOPP concentrations were not significantly associated with the sarcopenia classification under investigation [29], or were found to be significantly elevated in both breast cancer groups relative to healthy controls regardless of exercise status [22].

Inflammatory and cytokine mediators were reported in four studies [19,22,23,24], most frequently interleukin (IL)-6 [19,22], IL-10 and IL-1β [23], tumor necrosis factor-alpha (TNF-α) and interferon-gamma (IFN-γ) [24]. The direction of change for IL-6 differed between the two studies that assessed this marker: one trial reported a significant decrease in the exercise group [19], and an increase in the control group, whereas another reported an increase in IL-6 following resistance training [22], which the original authors interpreted in the context of an exercise-related inflammatory response. In the yoga-based trial, both IL-10 and IL-1β decreased significantly in the intervention group [23]. A separate report from the same yoga trial found significant reductions in TNF-α and IFN-γ following the intervention [24]. Given the limited number of studies, differences in cytokine panels, treatment settings, and sampling schedules, these findings should be interpreted descriptively rather than as evidence of a consistent anti-inflammatory effect of exercise.

Nitrosative stress markers were assessed in two reports [22,24]; S-nitrosothiols, peroxynitrite, and nitrotyrosine [22] were the least frequently assessed category, reported in only two studies [22,24]; in one, NO increased in the non-exercising control arm but remained essentially stable in the exercise arm [24], while in the other, several nitrosative markers, including peroxynitrite and nitrotyrosine, were significantly higher specifically in the supervised-exercise group [22], again raising the possibility that exercise-associated increases in redox turnover were not, in this particular clinical context, synonymous with a net reduction in oxidative or nitrosative burden.

### 3.4. Biological Specimens and Analytical Methods

The 12 studies relied predominantly on peripheral blood as the source specimen, although the specific blood fraction sampled differed across trials. Plasma was used in three studies [19,21,26], serum was used in four studies [20,23,24,25], and isolated erythrocytes were used as an additional or primary matrix in two studies assessing enzymatic antioxidant activity [20,21]. Beyond peripheral blood, one study additionally isolated peripheral blood mononuclear cells (PBMCs) to quantify gene-expression changes in antioxidant and heat-shock-protein transcripts [19], one used urine as the sole specimen for an oxidative DNA damage marker [28], and one incorporated cross-sectional computed tomography imaging at the third lumbar vertebra to derive skeletal muscle indices alongside its plasma-based redox panel [29]. The studies therefore used several distinct biological matrices, including plasma, serum, erythrocytes, peripheral blood mononuclear cells, and urine.

Analytical methods were similarly heterogeneous. Colorimetric or spectrophotometric assays represented the most frequently used analytical approach, applied to superoxide dismutase and catalase activity [19,20,22,24,30], total antioxidant capacity or ferric-reducing power [19,25,26,27,29], lipid peroxidation products [19,20,24,25], and protein oxidative damage markers [26,27,29]. Commercial immunoassays were used to determine the selected markers of oxidative DNA damage and protein oxidation. For example, plasma 8-OHdG was measured by enzyme-linked immunosorbent assay (ELISA) in Yen et al. [27] and Delrieu et al. [30], and nitrotyrosine levels were similarly evaluated in another study [22]. Multiplex bead-based immunoassay platforms (Luminex-type technology) were used to quantify circulating cytokine panels in two studies [23,24], allowing the simultaneous measurement of several interleukins or pro-inflammatory mediators from a single sample aliquot. Reverse-transcription quantitative PCR was applied in a single study to assess PBMC gene expression [19], representing the only instance of a molecular, rather than protein-level, redox readout in this literature. Notably, one study departed from the colorimetric or immunoassay approaches used elsewhere by quantifying urinary 8-oxo-7,8-dihydro-2′-deoxyguanosine (8-oxo-dG) using high-performance liquid chromatography coupled with electrospray ionization mass spectrometry [28], a considerably more analytically specific technique than the antibody- or absorbance-based methods used for the same biomarker class in other studies; this methodological divergence may partly explain why direct numerical comparison of DNA-damage outcomes across studies is not straightforward.

For a subset of enzymatic and non-enzymatic antioxidant markers, the specific commercial kit or in-house protocol was explicitly named in the original report, whereas for others the analytical platform was described only in general terms (e.g., “standard biochemical assay”) without a fully specified reference method. This variability in reporting granularity, together with the differences in specimen type described above, suggests that meta-analytic pooling of absolute biomarker concentrations across the included studies would require considerable caution, even where the nominal biomarker name (e.g., “SOD activity” or “TAC”) appears identical on the surface.

### 3.5. Timing of Sample Collection Relative to Exercise and Treatment

Most included studies adopted a simple two-point sampling structure, collecting biospecimens at baseline and again at the end of the intervention period. A smaller subset extended this to three or more timepoints: one study sampled at baseline, three months, and six months over the course of a 24-week program [21], one added a post-intervention detraining assessment twelve weeks after training cessation [20], and the yoga-based trial collected serum at baseline and at 16, 32, and 48 weeks [23,24], representing the most extensively repeated sampling schedule among the included reports. In the ABLE trial [29,30], fasting blood samples were collected at baseline and at the end of the six-month intervention, processed in EDTA tubes, and stored at −80 °C until analysis. Although fasting status was reported, the interval between the final exercise session and blood collection was not clearly specified; therefore, the extent to which the endpoint values reflected chronic training adaptation rather than recent activity exposure cannot be determined.

Reporting of the interval between the most recent exercise session and the blood or urine draw was inconsistent and, for most studies, was not addressed. Only one study explicitly instructed participants to abstain from exhaustive exercise for 72 h before each blood draw, together with a 12 h overnight fast [24], an approach that appears intended to minimize the confounding influence of an acute exercise bout on measures meant to reflect chronic training adaptation. Several other studies specified an overnight or several-hour fast prior to sampling but did not report whether, or how long before sampling, participants had last exercised [19,20,22,29,30]; this omission makes it difficult to determine whether the biomarker values reported in these studies reflect a stable trained state or a residual acute response to a recent training session. For the comparative study conducted during trastuzumab therapy [22], the redox panel for the supervised-exercise group appears to have been assessed following the completion of the six-week program, compared cross-sectionally against a non-exercising breast cancer group and a healthy reference group; a clearly reported within-person baseline measurement for the exercise group itself could not be identified in the original report, which limits the extent to which the observed between-group differences can be attributed specifically to the training program rather than to pre-existing differences among the three cohorts.

The relationship between the timing of blood sampling and the concurrent cancer treatment cycle (e.g., proximity to the most recent chemotherapy infusion) was likewise seldom specified in detail. Several studies stated only that participants were undergoing chemotherapy, radiotherapy, hormonal therapy, or trastuzumab concurrently with the exercise program [19,20,29,30], without indicating where the sampling day fell within a given treatment cycle. This reporting gap is important because biomarker values may vary across the treatment cycle. Consequently, lack of alignment between sampling day and treatment cycle could contribute to variability that cannot readily be distinguished from an exercise-related effect. Overall, the timing dimension of the included evidence appears to be one of the less completely reported methodological features across this literature, a pattern that may partly account for some of the directionally inconsistent findings noted earlier.

### 3.6. Reporting Completeness of Exercise Prescription and Treatment-Related Contextual Factors

Completeness in describing the exercise prescription varied considerably across the included studies. Several reports provided a fully specified frequency–intensity–time–type (FITT) profile, including a quantitative intensity metric expressed as a percentage of heart rate maximum, heart rate reserve, or one-repetition maximum, together with a defined session structure and progression scheme [19,20,22,26,27]. In these studies, intensity was typically adjusted over the course of the program according to a prespecified periodization plan, and in one case was individually calibrated to each participant’s ventilatory or anaerobic threshold as determined by a preliminary graded exercise test [26], representing a comparatively rigorous approach to intensity prescription. By contrast, intensity was reported only in qualitative or descriptive terms, without a corresponding heart rate- or load-based value, in the self-administered exercise program for postoperative breast cancer patients [21] and in the yoga-based trial [23,24], where the sequence and approximate duration of individual asanas and breathing exercises were described but no conventional cardiovascular intensity metric was provided. The walking-based intervention in metastatic breast cancer prescribed dose primarily through an individualized daily step count target rather than through a heart rate-based intensity band [30], and while this represents a quantifiable and progressively adjusted exercise parameter, it is not directly comparable to the intensity metrics used in the heart rate-based studies. The intensity bands distinguishing the moderate- and high-intensity arms of the colorectal cancer trial were named descriptively [28], but the specific heart rate or perceived exertion thresholds used to operationalize “moderate” versus “high” intensity were not clearly specified in the material available for this review, which limits replicability of this particular comparison.

Adherence to the prescribed program was quantified and reported as a numerical value in three studies [19,20,23], with completion rates in the 75–90% range among participants retained through study completion; one study applied a minimum adherence threshold of greater than 75% of sessions for inclusion in the final analysis [23]. Adherence was not reported as a discrete quantitative figure in the remaining studies [26,28,29,30], including those relying on unsupervised or self-directed exercise formats [21,30], where compliance was instead inferred from periodic self-report or activity-tracker data without a summary adherence percentage.

The reporting of the concurrent cancer treatment regimen was similarly uneven. Several studies specified the chemotherapeutic agents or regimen categories received by participants, either by naming individual drug combinations or by describing broader pharmacological classes such as platinum-coordinating complexes, alkylating agents, and antimetabolites [19,20,29,30], and another specified the endocrine agent (tamoxifen) together with its duration of use [20]. In contrast, several other studies described the concurrent treatment only in categorical terms—for example, as “chemotherapy and/or radiotherapy” or “hormonal therapy”—without further detail on specific agents, doses, or treatment schedules [21,25,26,28]. Disease stage was reported using a recognizable staging system (e.g., TNM or an equivalent categorical scheme) in approximately half of the included studies [19,20,23,24,27], whereas the remainder either did not report a formal stage classification or reported it only implicitly through the trial’s eligibility criteria (e.g., “metastatic” or “following primary treatment”) [25,26,28,29,30]. This pattern indicates that exercise-prescription variables, adherence, and treatment-related contextual details were reported with varying levels of completeness across the included literature, and that no single study reported all of these dimensions with the same degree of specificity. These differences reflect variation in reporting practice and should not be interpreted as a judgment of the quality of individual studies.

## 4. Discussion

**Figure 2:**
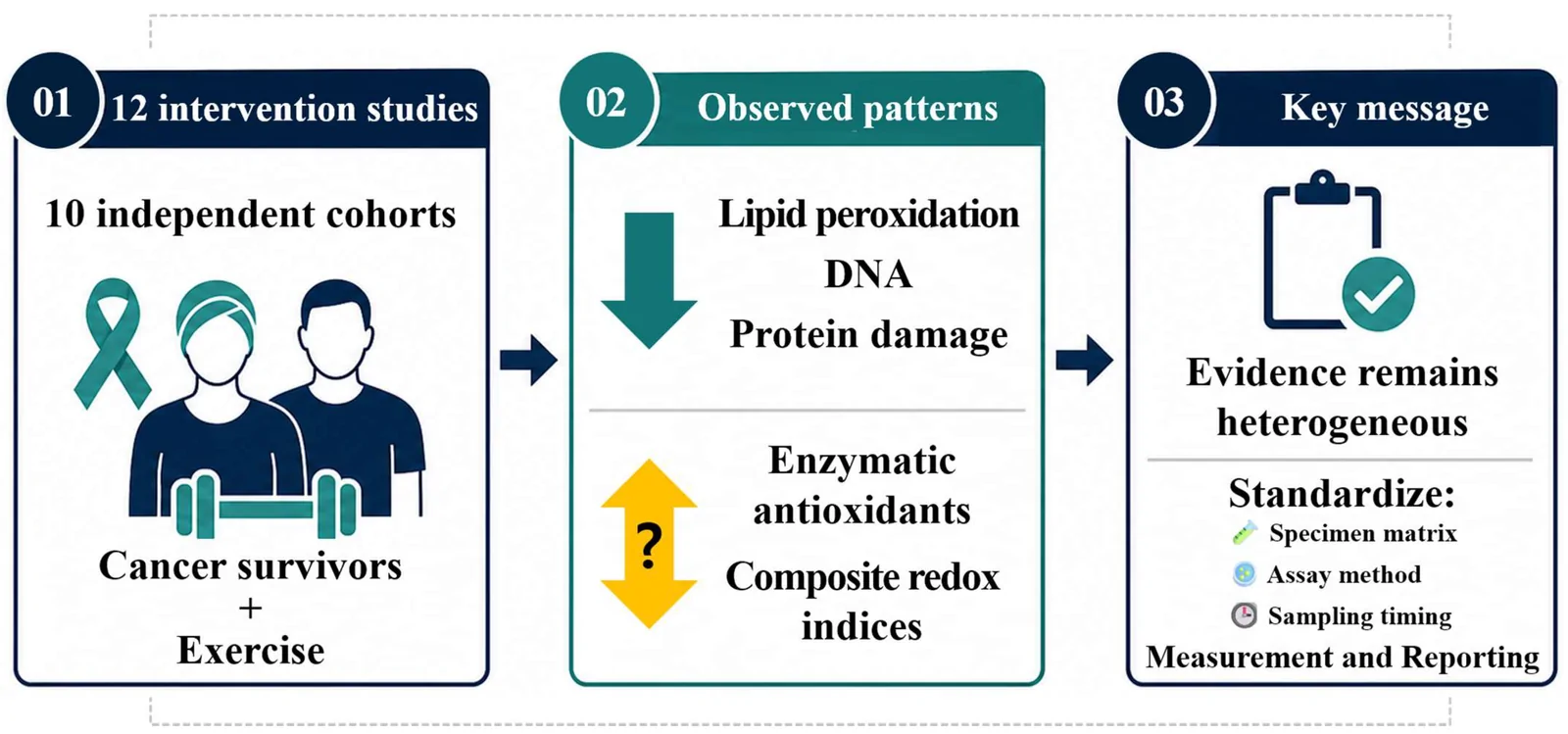
Redox biomarker patterns and methodological heterogeneity in exercise oncology studies.

This scoping review mapped the measurement and reporting characteristics of oxidative stress and antioxidant biomarkers in exercise intervention studies involving adults with cancer. The included reports differed substantially in biomarker selection, biological matrix, analytical platform, normalization procedure, biospecimen collection schedule, exercise prescription, and treatment-related context (Figure 2). Accordingly, the descriptive patterns in biomarker direction reported in the Results should not be interpreted as pooled evidence of intervention efficacy.

### 4.1. Specimen and Analytical Method Heterogeneity

Interpretation of redox biomarkers depends not only on the nominal biomarker selected, but also on the biological matrix, analytical platform, pre-analytical handling, and basis used to express the result. The variation in specimen matrices and analytical approaches documented in the present review should therefore be considered an important limitation when comparing direction or magnitude of change across studies, rather than a minor technical difference.

For antioxidant enzymes, the biological meaning of a measured activity is likely to depend substantially on the specimen in which it is assessed. Superoxide dismutase and catalase are predominantly intracellular enzymes, and measurements obtained from erythrocytes, plasma, or serum would not necessarily be expected to reflect the same biological compartment or physiological process [8,31,32]. Extracellular concentrations or activity of redox enzymes may also be influenced by factors such as cell damage, hemolysis, secretion, and sample processing, which further complicates direct comparison with erythrocyte-based measures [32]. Accordingly, apparently discordant responses of the same nominal enzyme across studies should not be interpreted as evidence of inconsistent biological adaptation without considering the specimen matrix and the accompanying analytical procedure. Analytical specificity is also relevant when interpreting markers of lipid peroxidation and oxidative DNA damage. The thiobarbituric acid reaction is not molecularly specific for malondialdehyde, because thiobarbituric acid can react with several non-lipid carbonyl-containing compounds, and the resulting signal may be influenced by sample storage and handling conditions [33]. Similarly, measurement of 8-OHdG requires particular caution because interlaboratory studies have shown substantial variation in estimates obtained using different analytical procedures [34,35,36]. Chromatographic approaches with appropriate quality-control procedures generally provide greater analytical specificity than methods that rely solely on antibody recognition or absorbance, although method performance remains dependent on the full analytical workflow [34,35,36]. Differences in normalization procedures provide an additional barrier to quantitative comparison. Enzyme activity expressed relative to hemoglobin, total protein, plasma volume, or another denominator cannot be assumed to be numerically interchangeable unless the relevant conversion data and methodological details are available. Future exercise-oncology trials would therefore benefit from prespecifying the specimen matrix, sample-processing procedures, assay platform, calibration and quality-control procedures, and normalization approach for each redox endpoint. Greater consistency in these elements would improve the interpretability of individual studies and facilitate more meaningful evidence synthesis.

### 4.2. Sampling Timing and Alignment with the Treatment Cycle

Temporal standardization is particularly important when redox biomarkers are used to evaluate a chronic exercise intervention. A single exercise bout can induce marker-specific responses that emerge and resolve over different time courses. In a time-course study of strenuous aerobic exercise, catalase activity, TBARS, total antioxidant capacity, and protein carbonyls showed peak responses at different post-exercise intervals, indicating that the biological interpretation of a blood sample may depend materially on the interval between the last exercise session and sample collection [6]. Although the magnitude and timing of these responses may differ in patients with cancer, the same principle suggests that biomarker values obtained soon after exercise may not represent the same physiological state as values obtained after an adequately standardized recovery interval. The timing of sampling in relation to cancer treatment may introduce an additional source of temporal variation. Cytotoxic chemotherapy, radiotherapy, endocrine therapy, and targeted therapies may each alter redox homeostasis through distinct mechanisms, and treatment-related changes may vary across a treatment cycle [7,13]. For example, longitudinal observations in patients receiving chemotherapy have indicated that glutathione-related markers and antioxidant enzyme activities may differ between pre-treatment and post-cycle assessments [7]. Although such findings cannot be generalized directly across all cancer types and treatment regimens, they support the need to document the interval between biospecimen collection and the most recent treatment administration. For future trials, a sampling protocol should specify, at minimum, the fasting requirement, the permitted level of physical activity before sampling, the interval from the final exercise session, the time of day, and, where applicable, the treatment cycle day and interval from the most recent treatment administration. Repeated measurements at standardized intervals may be especially informative where the aim is to distinguish acute exercise responses from longer-term training adaptation. Without such information, differences in biomarker values may reflect a combination of training-related adaptation, recent exercise exposure, treatment-related fluctuation, and pre-analytical variation.

During active cancer treatment, the interpretation of redox biomarkers requires particular caution because exercise-related signals may occur against a background of treatment-induced oxidative and inflammatory perturbation [9,26]. Chemotherapy and radiotherapy may increase reactive oxygen species generation through mechanisms relevant to their antitumor effects [9], whereas treatment-related fatigue, reduced dietary intake, altered body composition, inflammation, and reduced physical activity may also influence systemic redox status [26]. The direction and magnitude of a biomarker response may therefore depend not only on the planned exercise dose, but also on treatment modality, cumulative dose, cycle day, disease burden, and the interval between the final exercise session and biospecimen collection [37,38]. In this context, an increase in an oxidant-related marker or antioxidant enzyme activity should not automatically be interpreted as either a harmful response or a beneficial hormetic adaptation. Rather, these findings need to be considered alongside the assay used, the sampled compartment, the treatment timeline, and the study design.

### 4.3. Incomplete Reporting of Exercise Prescription and Treatment Context

The variation in exercise-prescription and treatment-context reporting identified in the present review appears consistent with a broader reporting problem in exercise oncology. In a systematic review of 138 exercise interventions from 131 oncology randomized controlled trials, no item of the Template for Intervention Description and Replication (TIDieR) checklist was reported completely across all interventions, and reporting of exercise-provider expertise or training was particularly limited [39]. Information concerning intervention adherence was also reported in fewer than half of the interventions assessed [39]. These findings suggest that incomplete reporting is not confined to redox-focused exercise studies but may limit reproducibility across the wider exercise-oncology literature. This issue is especially relevant for redox outcomes because the physiological stimulus produced by exercise depends on dose, progression, adherence, supervision, and individual tailoring. When these features are insufficiently described, a null or heterogeneous biomarker response cannot readily be distinguished from a response to an exercise dose that differed materially from that intended by the investigators. Similarly, incomplete characterization of concurrent cancer treatment limits interpretation because treatment-related oxidative burden may differ according to treatment modality, regimen, dose, and timing [7]. These considerations do not establish that reporting limitations explain the observed variability in biomarker outcomes; rather, they define important sources of uncertainty that should be considered when interpreting the current evidence base. Future exercise-oncology trials incorporating redox biomarkers should report intervention content using the TIDieR checklist [40] together with the Consensus on Exercise Reporting Template [41]. In addition to modality, frequency, session duration, intensity, progression, supervision, and adherence, reports should provide sufficient information on the exercise provider, delivery setting, modifications, adverse events, concurrent treatment regimen, and treatment timing to permit replication and to support interpretation of biomarker data. More complete reporting would not eliminate biological heterogeneity, but it would enable future reviews to examine whether differences in exercise dose or treatment context contribute to between-study variation.

To translate these considerations into a practical resource, Table 4 presents a proposed reporting checklist for redox biomarker studies in exercise oncology. The checklist integrates core elements of exercise reporting, treatment context, biospecimen collection, pre-analytical handling, analytical procedures, and data expression. Its purpose is not to prescribe a single exercise program or biomarker protocol, but to identify the minimum information needed to support the interpretation, replication, and comparison of redox biomarker findings.

The reporting considerations in Table 4 were derived from the measurement and reporting limitations mapped in the present review. They are not a validated consensus guideline and do not prescribe a single optimal biomarker panel, specimen matrix, assay platform, exercise program, or post-exercise sampling interval. Rather, they identify information that should be reported to allow redox biomarker findings to be interpreted in relation to the exercise stimulus, treatment context, biospecimen handling, and analytical method, and to support replication and comparison across studies.

### 4.4. Cancer Type and Treatment Context in the Interpretation of Redox Biomarkers

The clinical context in which exercise is delivered should be considered when interpreting redox biomarker findings. Although the present review included patients during active chemotherapy, radiotherapy, endocrine therapy, trastuzumab treatment, and post-treatment survivorship, the evidence base was dominated by women with breast cancer and contained too few reports within individual cancer treatment combinations to support treatment-specific conclusions (Table 3). The observed heterogeneity should therefore not be interpreted as evidence that exercise produces a uniform redox response across cancer populations. Patients receiving cytotoxic chemotherapy may experience treatment-related oxidative stress, inflammation, mitochondrial dysfunction, fatigue, and reductions in physical capacity that could influence both the physiological response to exercise and the biomarker values measured during an intervention [7,10]. The magnitude and timing of these effects are likely to depend on the specific agent, cumulative dose, treatment schedule, supportive medication, and interval between treatment administration and biospecimen collection [7]. Radiotherapy may also contribute to oxidative DNA damage and treatment-related redox disturbance; however, the extent to which circulating biomarkers reflect these effects may vary according to radiation exposure and other clinical factors [2]. Evidence concerning endocrine therapy and targeted therapy was limited to individual breast cancer studies in the present review; these treatment settings should therefore not be assumed to represent the same acute redox context as cytotoxic chemotherapy [20,22]. These distinctions are particularly important because circulating oxidative stress and antioxidant biomarkers do not identify the tissue source of the measured signal [8]. A change in plasma, serum, erythrocyte, or urinary biomarker concentration during exercise training may reflect exercise-related adaptation, treatment-related redox effects, disease-related processes, pre-analytical variation, or a combination of these influences [8,9]. Future trials should therefore report cancer type, disease stage, treatment intent, specific treatment regimen, treatment dose or cycle number, and the interval between the most recent treatment administration and biospecimen collection. Where sample size permits, analyses should also be stratified a priori according to treatment phase and treatment modality rather than combining active-treatment and post-treatment populations in a single estimate.

The concentration of evidence in women with breast cancer further limits the scope of inference. Nine of the 12 included reports involved breast cancer populations, and several were conducted during endocrine therapy [20,22], trastuzumab treatment [22], or breast cancer-directed chemotherapy [23,24] or radiotherapy [24]. These clinical contexts differ from those of hematological malignancies, gastrointestinal cancers, lung cancer, prostate cancer, and other tumor types with respect to treatment exposures, symptom burden, nutritional status, body composition changes, and exercise tolerance. The present findings should therefore be regarded as a map of the available measurement and reporting practices, primarily within breast cancer research, rather than as evidence that can be generalized across cancer diagnoses, sexes, or treatment regimens.

### 4.5. Limitations of the Present Review and Recommendations for Future Research

This review has several limitations. Although the protocol was registered on the OSF, the search was limited to English-language publications and four databases. In addition, supplementary searches of trial registries and conference proceedings were not conducted. Therefore, some eligible studies, particularly those published in languages other than English, may have been missed. As with any review of peer-reviewed literature, publication bias cannot be excluded: trials that failed to detect a redox effect or were discontinued for feasibility reasons may be under-represented relative to those reporting a positive finding. A more consequential limitation concerns the deliberate decision not to conduct a formal risk-of-bias assessment, consistent with scoping review methodology. Its practical effect is that the patterns described above cannot be weighted by methodological rigor, so a small unblinded pilot study has contributed to this synthesis on the same footing as a larger randomized trial. Consistent with the scoping purpose of this review, the present findings characterize the available evidence and should not be interpreted as a bias-adjusted estimate of intervention effects or as an assessment of certainty of evidence. A future systematic review applying RoB 2 to randomized trials and ROBINS-I to non-randomized studies would be needed to address these questions.

The available evidence also has important limitations. Sample sizes were generally small, ranging from 12 to 130 participants, and several reports were explicitly framed as pilot or feasibility work. In addition, four reports arose from two underlying cohorts: the two reports by Delrieu et al. [29,30]; and the two reports by Jain et al. [23,24]. These paired publications contributed distinct outcome information but should not be interpreted as evidence from four independent cohorts. The population was also skewed toward women with breast cancer, so applicability to other cancer types, male patients, or older and more comorbid populations remains uncertain. Future updates would benefit from broader searches that include trial registries and the non-English literature. At the primary-research level, the heterogeneity discussed above suggests the field may benefit from coordinated biomarker, specimen, and sampling timing selection; a stakeholder-informed process analogous to core outcome set development, as described in the COMET Handbook [45], offers one plausible mechanism, though it has not previously been applied to redox biomarkers in exercise oncology. Larger trials with a priori power calculations for the redox endpoint itself, rather than for a co-primary functional outcome, would also strengthen the evidentiary basis for future recommendations.

## 5. Conclusions

In summary, the 12 exercise intervention reports charted in this review described reductions in lipid peroxidation and selected protein or DNA oxidative damage markers in several studies, whereas enzymatic antioxidants and composite redox indices showed directionally mixed responses. These patterns should be interpreted cautiously because the included studies varied substantially in cancer type, treatment phase, treatment context, exercise prescription, biological specimen, analytical platform, normalization procedure, and sampling timing. The evidence base was heavily weighted toward women with breast cancer, and the small number of reports involving other cancer types or specific treatment settings precludes cancer-specific or treatment-specific conclusions. In particular, redox biomarker findings obtained during active chemotherapy, radiotherapy, endocrine therapy, or targeted treatment should not be assumed to represent the same biological context as findings obtained during post-treatment survivorship. To support future research, this review provides a proposed reporting checklist for redox biomarker studies in exercise oncology. The checklist emphasizes the reporting of exercise dose and adherence, cancer treatment regimen and timing, biospecimen collection and processing procedures, analytical methods, normalization approaches, and the interval between the most recent exercise session, treatment administration, and biomarker sampling. Although the checklist is not a validated consensus guideline, it provides a practical starting point for improving the interpretability, reproducibility, and comparability of future studies. Until greater methodological consistency is achieved, meta-analytic pooling of redox biomarkers in exercise-oncology research should be interpreted with caution.

## Figures and Tables

**Table 1:** PCC-based eligibility criteria.

Component	Inclusion	Exclusion
Population	Adults (≥18 years) diagnosed with any type of malignancy, at any stage of active treatment or in post-treatment survivorship	Pediatric or adolescent populations (<18 years); healthy individuals without a cancer diagnosis; animal or in vitro models
Concept	A structured exercise intervention (aerobic, resistance, combined, or mixed-modality) reporting at least one biomarker intended to reflect oxidative damage, antioxidant defense, global oxidant/antioxidant balance, or nitrosative stress. Eligible biomarkers included lipid peroxidation products, oxidative DNA damage markers, protein oxidation markers, enzymatic or non-enzymatic antioxidant defenses, total oxidant or antioxidant capacity measures, and nitrosative stress markers	Studies assessing physical activity only through self-report questionnaires without an exercise intervention component; studies reporting only inflammatory, metabolic, functional, or quality-of-life outcomes without at least one eligible redox biomarker
Context	Any healthcare, community, home-based, or research setting; any country; no restriction on cancer type or treatment modality	Studies combining exercise with a co-intervention (e.g., pharmacological antioxidant supplementation) in which the exercise-specific effect on biomarkers could not be distinguished
Study design	Randomized controlled trials, non-randomized controlled trials, single-arm pre–post intervention studies, and prospective cohort studies with an exercise exposure	Narrative reviews, systematic reviews, meta-analyses, case reports, conference abstracts without a retrievable full text, and study protocols without outcome data
Publication type	Peer-reviewed original research articles	Editorials, letters, and gray literature
Language	English-language publications	Non-English publications, owing to translation resource constraints

**Table 2:** Database-specific search strategies.

Database	Search String
PubMed/ MEDLINE	(“neoplasms”[MeSH Terms] OR cancer*[tiab] OR oncolog*[tiab] OR tumor*[tiab] OR carcinoma*[tiab] OR malignan*[tiab] OR “cancer survivor*”[tiab]) AND (“exercise”[MeSH Terms] OR “exercise therapy”[MeSH Terms] OR exercise*[tiab] OR “physical activity”[tiab] OR “resistance training”[tiab] OR “aerobic exercise”[tiab] OR “exercise intervention”[tiab] OR “physical exercise”[tiab]) AND (“oxidative stress”[MeSH Terms] OR “reactive oxygen species”[MeSH Terms] OR “antioxidants”[MeSH Terms] OR oxidative[tiab] OR “lipid peroxidation”[tiab] OR malondialdehyde[tiab] OR “8-hydroxy-2′-deoxyguanosine”[tiab] OR “8-OHdG”[tiab] OR “superoxide dismutase”[tiab] OR glutathione[tiab] OR “total antioxidant capacity”[tiab] OR “redox status”[tiab])
Embase	(‘neoplasm’/exp OR cancer*:ti,ab OR oncolog*:ti,ab OR tumor*:ti,ab OR carcinoma*:ti,ab OR malignan*:ti,ab OR ‘cancer survivor*’:ti,ab) AND (‘exercise’/exp OR ‘physical activity’/exp OR exercise*:ti,ab OR ‘physical activity’:ti,ab OR ‘resistance training’:ti,ab OR ‘aerobic exercise’:ti,ab OR ‘exercise intervention’:ti,ab) AND (‘oxidative stress’/exp OR ‘reactive oxygen species’/exp OR ‘antioxidant’/exp OR oxidative:ti,ab OR ‘lipid peroxidation’:ti,ab OR malondialdehyde:ti,ab OR ‘8 hydroxy 2 deoxyguanosine’:ti,ab OR ‘8-ohdg’:ti,ab OR ‘superoxide dismutase’:ti,ab OR glutathione:ti,ab OR ‘total antioxidant capacity’:ti,ab OR ‘redox status’:ti,ab)
Scopus	TITLE-ABS-KEY((cancer* OR oncolog* OR tumor* OR carcinoma* OR malignan* OR “cancer survivor*”) AND (exercise* OR “physical activity” OR “resistance training” OR “aerobic exercise” OR “exercise intervention”) AND (oxidative OR “reactive oxygen species” OR antioxidant* OR “lipid peroxidation” OR malondialdehyde OR “8-hydroxy-2′-deoxyguanosine” OR “8-OHdG” OR “superoxide dismutase” OR glutathione OR “total antioxidant capacity” OR “redox status”))
Web of Science Core Collection	TS = (cancer* OR oncolog* OR tumor* OR carcinoma* OR malignan* OR “cancer survivor*”) AND TS = (exercise* OR “physical activity” OR “resistance training” OR “aerobic exercise” OR “exercise intervention”) AND TS = (oxidative OR “reactive oxygen species” OR antioxidant* OR “lipid peroxidation” OR malondialdehyde OR “8-hydroxy-2′-deoxyguanosine” OR “8-OHdG” OR “superoxide dismutase” OR glutathione OR “total antioxidant capacity” OR “redox status”)

*: A truncation wildcard used to search for words with different endings.

**Table 3:** Experimental studies on exercise and redox biomarkers in adult cancer patients and survivors.

Study	Participants/Model	Intervention	Measures	Results
Moulton et al. 2023 [19]	40 post surgery female BC patients, adjuvant therapy	16-week online home-based mixed EX; structured sessions 3×/week	- Plasma: SOD, CAT, TBARS, TAC, total free thiol - PBMC: SOD2 mRNA expression	- SOD, CAT ↑ - TBARS ↓ - TAC ↑, total free thiol ↑ - SOD2 mRNA ↑
Repka and Hayward 2018 [26]	15 cancer pts (EX *n* = 8, CON *n* = 7) + 7 healthy controls	10-week supervised aerobic + resistance EX, 3×/week	- Plasma: 8-OHdG, protein carbonyl, TAC - Symptom: cancer-related fatigue	- TEAC ↑ - Protein carbonyl ↓ - 8-OHdG—(but favorable EX vs. CON interaction) ↓ - Fatigue ↓
de Jesus Leite et al. 2021 [20]	32 BC survivors on tamoxifen	12-week supervised EX (aerobic + resistance) followed by 4-week detraining	- Serum: MDA, SOD, CAT, GPx - Body composition, muscle strength	- MDA ↓ - SOD, CAT, GPx ↑ - Favorable changes in strength and body composition
Feifei Li et al. 2021 [21]	130 BC pts post-op/chemo (*n* = 65 EX, *n* = 65 CON)	24-week self-controlled EX, 4×/week	- RBC SOD, GSH-Px, plasma MDA - Joint mobility - 6MWD	- SOD ↑ at 3 mo, ↓ at 6 mo - GSH-Px – - MDA ↓ - Joint mobility, 6MWD ↑
Delrieu et al. 2021 [29]	49 women with metastatic BC	6-month individualized PA intervention, single-arm	- Plasma: MDA, protein carbonyl, TAC - Sarcopenia indices	- TAC ↑ - MDA, protein carbonyl – - Muscle function tended to improve
Delrieu et al. 2021 [30]	49 women with metastatic BC	6-month individualized walking intervention with an activity tracker	- Plasma: 8-OHdG, MDA, AOPP, SOD, CAT, GPx, MPO - Leukocytes: NOX, MPO	- GPx, MDA ↑ - 8-OHdG, SOD, CAT – - NOX, MPO –
Allgayer et al. 2008 [28]	48 patients with colorectal carcinoma after primary treatment	2-week moderate-intensity EX vs. high-intensity EX vs. no EX	- Urinary 8-oxo-dG	- Moderate EX: 8-oxo-dG ↓ - High-intensity EX, Control: 8-oxo-dG –
Yen et al. 2020 [27]	30 patients with head and neck cancer undergoing chemotherapy	8-week combined aerobic + resistance exercise, 3×/week	- Plasma: TAC, 8-OHdG, MDA, protein carbonyl - Exercise tolerance (BP/HR)	- TAC ↑ - 8-OHdG, protein carbonyl ↓ - MDA – - Exercise tolerance ↑
Jain et al. 2022 [23]	96 stage II–III BC pts, chemo/radio	48-week yoga, 5×/week vs. non-yoga	- Serum: IL-10, IL-1β - Cancer-related fatigue - Functional scales	- IL-10 ↓ - IL-1β ↓ - Fatigue ↓ - Functional scale ↑
Jain et al. 2023 [24]	96 stage II–III BC pts, chemo/radio	48-week yoga, 5×/week vs. non-yoga	- Serum SOD, CAT, MDA, NO - TNF-α, IFN-γ - QoL	- MDA ↓ - SOD/CAT – - TNF-α, IFN-γ ↓ - QoL ↑
Domaszewska et al. 2021 [25]	12 BC survivors, single-arm pilot	8-week endurance training (cycle ergometer), 3×/week	- Serum: FRAP (total antioxidant capacity), TBARS (lipid peroxidation)	- FRAP – - TBARS –
Hojan et al. 2022 [22]	50 HER2 + BC pts on trastuzumab (BC *n* = 25, BC + PE *n* = 25) + 50 healthy controls	6-week supervised aerobic + resistance EX (BC + PE), 5×/week vs. usual-activity BC group	- Serum SOD, CAT, GSH; TAC, TOS, OSI - LOOH, AOPP - NO, S-nitrosothiols	- SOD, CAT, TAC, TOS, OSI ↑ in BC + PE - GSH ↓ in both BC groups - LOOH, AOPP, NO higher in both BC groups vs. CG

BC: Breast cancer; EX: Exercise; SOD: Superoxide dismutase; CAT: Catalase; GPx/GSH-Px: Glutathione peroxidase; MDA: Malondialdehyde; TBARS: Thiobarbituric acid-reactive substances; TAC/TEAC: Total antioxidant capacity; FRAP: Ferric-reducing antioxidant power; TOS: Total oxidant status; OSI: Oxidative stress index; 8-OHdG/8-oxo-dG: DNA oxidation markers; LOOH/AOPP: Lipid/protein oxidative damage; NO: Nitric oxide; NOX: NADPH Oxidase; MPO: Myeloperoxidase; PBMC: Peripheral blood mononuclear cells; IL: Interleukin; 6MWD: 6 min walk distance; No significant: –; Significant increase or decrease: ↑↓.

**Table 4:** Pragmatic reporting considerations for redox biomarker assessment in exercise-oncology intervention studies.

Domain	Minimum Item to Report	Practical Recommendation and Rationale	Ref.
Participant and disease characteristics	Cancer type, histological subtype where relevant, disease stage, sex, age, treatment intent, and time since diagnosis	These characteristics should be reported separately for each study group because baseline redox status and exercise tolerance may differ across cancer populations.	[42]
Cancer treatment context	Current treatment status; specific treatment modality; drug name or regimen; cumulative dose or cycle number where available; radiotherapy field, dose, and fractionation where applicable	Treatment-related oxidative burden should not be inferred from broad labels such as “chemotherapy” or “radiotherapy” alone.	[7]
Timing relative to treatment	Date or cycle day of the most recent treatment administration and the interval between treatment and biospecimen collection	This information is particularly important during active treatment because biomarker values may vary across the treatment cycle.	[7]
Exercise intervention	Exercise modality, frequency, session duration, program duration, exercise intensity metric, progression rule, supervision, setting, and individualization procedures	Intensity should be operationalized using a reproducible metric, such as heart rate reserve, percentage of one-repetition maximum, oxygen uptake, power output, or rating of perceived exertion.	[41]
Exercise dose received	Number and proportion of completed sessions, reasons for missed sessions, modification of planned exercise, adverse events, and actual exercise dose achieved	Planned dose and completed dose should be distinguished to allow interpretation of adherence and intervention fidelity.	[40,41]
Pre-sampling exercise exposure	Exact interval between the final exercise session and biospecimen collection; restrictions on strenuous exercise before sampling	For studies intended to assess chronic training adaptation, investigators should prespecify and report a standardized exercise-abstention interval. A universal optimal interval cannot yet be established from the available evidence.	[6]
Sampling conditions	Time of day, fasting duration, hydration instructions, medication restrictions where relevant, menstrual or menopausal status where relevant, and acute illness at sampling	These factors may influence circulating biomarker concentrations and should be standardized or documented.	[43]
Biospecimen characteristics	Specimen matrix, anticoagulant or collection tube, processing interval, centrifugation procedure, storage temperature, storage duration, and freeze–thaw history	Plasma, serum, erythrocytes, peripheral blood mononuclear cells, and urine should not be treated as interchangeable matrices.	[43]
Biomarker selection	Biomarker name, biological rationale, oxidative stress domain, and whether the biomarker represents oxidative damage, antioxidant capacity, enzymatic activity, or redox-related signaling	The interpretation of each biomarker should be aligned with the biological compartment and analytical method used.	[8,44]
Analytical procedure	Assay platform, kit manufacturer and catalog number where relevant, calibration procedure, intra- and inter-assay coefficient of variation, detection limit, and quality-control method	Such information is necessary for interpretation, replication, and comparison across studies.	[8,43]
Data expression	Unit of measurement, normalization basis, handling of values below the limit of detection, and treatment of missing biomarker data	Enzyme activity normalized to hemoglobin, total protein, plasma volume, or another denominator should not be assumed to be numerically interchangeable.	[8]
Analysis and reporting	Prespecified primary redox endpoint, timing of outcome assessment, treatment-adjusted analysis where feasible, and reporting of both effect estimates and uncertainty	Reporting should permit readers to distinguish exploratory biomarker findings from prespecified study outcomes.	[40,42]

## Data Availability

The data charting form and full extracted dataset are available from the corresponding author upon reasonable request.

## Abbreviations

The following abbreviations are used in this manuscript:

ROS	reactive oxygen species
8-OHdG	8-hydroxy-2′-deoxyguanosine
MDA	malondialdehyde
TBARS	thiobarbituric acid-reactive substances
SOD	superoxide dismutase
CAT	catalase
TIDieR	Template for Intervention Description and Replication
GPx	glutathione peroxidase
TAC	Trolox-equivalent antioxidant capacity
FRAP	ferric-reducing antioxidant power
TOS	total oxidant status
OSI	oxidative stress index
AOPP	advanced oxidation protein products
IL	interleukin
TNF-α	tumor necrosis factor-alpha
IFN-γ	interferon-gamma
NO	nitric oxide
